# Personal Variables of Protection against Cannabis Use in Adolescence: The Roles of Emotional Intelligence, Coping Styles, and Assertiveness as Associated Factors

**DOI:** 10.3390/ijerph18115576

**Published:** 2021-05-23

**Authors:** Sara González-Yubero, Susana Lázaro-Visa, Raquel Palomera

**Affiliations:** Education Faculty, University of Cantabria, 39005 Santander, Spain; susana.lazaro@unican.es (S.L.-V.); raquel.palomera@unican.es (R.P.)

**Keywords:** emotional intelligence, coping styles, assertiveness, cannabis, adolescence, prevention

## Abstract

(1) Background: Many public bodies have warned of the increased consumption of cannabis, particularly among adolescents. From the Positive Youth Development approach, the promotion of personal protective factors takes on special importance against some risks, such as the consumption of addictive substances. (2) Methods: This research is one of the first to study the role of trait and ability emotional intelligence in relation to cannabis use and with respect to other personal variables of protection, such as coping styles and assertiveness. For this purpose, a final sample of 799 schoolchildren was obtained. (3) Results: After controlling for age and gender, the results of the regression analyses revealed that emotional perception, emotional facilitation, emotional clarity, emotional repair, active coping style, and assertiveness were inversely and significantly associated with cannabis use behaviors. On the other hand, the emotional attention and avoidant coping style factors were positively and significantly associated with these behaviors. (4) Conclusions: These findings provide new evidence that could be useful in terms of guiding health-promoting clinical and educational interventions at an early age.

## 1. Introduction

Cannabis use in the adolescent population is currently a serious public health concern [1]. In the latest report from the European Monitoring Center for Drugs and Addiction [2], it is estimated that 17.5 million (14.4%) young Europeans aged between 15 and 34 used cannabis in the last year. Regarding the situation in Spain, cannabis is the most-used illegal psychoactive substance among students aged 14 to 18: 33% of Spanish adolescents are reported to have consumed this substance at some point in their lives; 27% consumed it during the last year; 15.4% reported problematic cannabis use which is related to the excessive amount of consumption, consumption at the offer of friends, etc. [3]. The latest Spanish Survey on Drug use in Secondary School Students (ESTUDES) [3] shows that the average age at which cannabis use starts is around 14–15 years. The frequency and quantity of cannabis consumption increases with age and its use is more common among men than women.

Various studies have highlighted that peer group cannabis consumption is one of the main causes of the use of this substance among adolescents [4,5]. Likewise, adolescents who begin cannabis consumption at an early age are more susceptible to consuming other illegal substances and developing a pattern of risky alcohol consumption [4]. Previous studies warn that cannabis consumption has been associated with the appearance of mental disorders among adolescents [6,7]. The use of this substance has also been associated with increased incidence of academic problems, conflicts or physical aggression at school [8], behaviors related to gambling and problematic internet use [9,10], sexual abuse [11], episodes of anxiety and depression, acts of suicide [12,13], and greater probability of dependency in adult life [14]. The seriousness of these consequences highlights the need to reinforce preventive strategies from an early age [15].

From the Positive Youth Development model a perspective focused on personal protective factors for healthy development is adopted, which includes the necessary skills to face diverse risks such as the use of addictive substances [16]. In this sense, coping styles deserve particular attention [17], as well as assertive behavior, which is considered a fundamental element of the socialization process [18]. To these factors a new beneficial construct for healthy development called Emotional Intelligence (EI) is added, whose promotion at an early age could represent a novel preventive approach [19]. Given that cannabis use begins at an increasingly young age [20], this research aims to study the influence of these personal factors on the use of this substance in adolescence.

Coping styles play an important mediating role between stressful situations, adolescents’ resources to deal with them, and the resulting consequences for their physical and mental health [21]. This moderating function is especially relevant in this complex period of life in which important changes are experienced [22]. Following Ebata and Moos [23], it is possible to distinguish between an active coping style (planning on ways to solve a problem, cognitive reassessment, social support, etc.) that facilitates the adolescent’s adjustment, and a dysfunctional or avoidant coping style (focused on avoiding or denying problems or delegating solutions to external factors) that increases the probability of psychosocial problems. Some studies with adolescents have found that less use of active coping has been related to an increase in the probability of ever consuming cannabis, as well as to doing so more frequently and in greater quantities [24,25]. Other studies suggest that the use of a dysfunctional coping style has been related to greater risk of consuming cannabis due to peer pressure [25,26,27]. The literature has confirmed a link between coping styles and drug use behaviors [17]. Despite this, the access to substances such as cannabis could be more complicated for adolescents as they are not financially independent, consequently, many of the studies have been carried out with adult samples [28] and would be relevant to analyse what happens in the adolescent stage.

In turn, one of the personal variables that is most related to the use of drugs in adolescents is assertive behavior [29]. Assertiveness implies acting in defense of one’s own interests, defending oneself without unjustified anxiety, sincerely and kindly expressing one’s feelings, and putting one’s personal rights into practice while respecting those of others [18]. Focusing on cannabis consumption, the effort dedicated to prevention is generally justified on the assumption that peer group influence is relevant in the initiation and maintenance of this habit [30]. Various studies have claimed that a deficit in assertive skills is associated with an increased risk in adolescents’ use of cannabis [10,31], as well as a lower capacity to oppose peer pressure when consuming it [32]. However, the consideration of assertive behavior as a substance use protection factor is contradicted in some empirical observations [33]. Therefore, this study aims to confirm whether assertiveness predicts cannabis use by adding extra empirical evidence when considering it as a central element in clinical and educational interventions.

Over the last two decades, interest in studying Emotional Intelligence (EI) has increased. EI is understood as “the ability to recognize, understand and regulate one’s own emotions and those of others, discriminate between them and use the information as a guide to thoughts and actions” [34] (p. 10). It should be noted that the scientific literature distinguishes two EI constructs that can be differentiated on the basis of the measurement method used to operationalize it [35]. On the one hand, self-reports measure trait EI through the perception of the person’s own emotional skills. On the other hand, the maximum performance tests of EI measure the cognitive ability to give correct or incorrect responses to emotional tasks. The trait EI construct belongs to the field of personality, while the second refers to cognitive ability, thus the scientific literature has been developed independently. The EI self-report measures have been widely used in research. However, maximum performance tests have been barely used athough the literature highlights the importance of applying them to assess the real capacity of people when solving emotional tasks [36].

In the field of drug use, data from a systematic review confirmed the association between lower EI and a more problematic use of alcohol, tobacco, cannabis, and other illegal substances mostly in adults [37]. Some research that evaluated trait EI found that university students who used cannabis were less able to understand and repair their negative emotional states and started consumption at an earlier age [38]. A higher perceived ability to regulate emotions was related to a lower frequency of cannabis use in other studies with adolescents [39,40]. On the other hand, a high level of attention to emotions has been linked to a higher level of stress, which hinders the ability to understand and regulate emotions and increases the probability of cannabis use in adolescents [41]. In terms of EI evaluated through maximum performance tests, the few existing studies such as the one carried out by Brackett, Mayer, and Warner [42], showed significant negative relations between the components of emotional perception, emotional facilitation, emotional understanding, and emotional regulation regarding the frequency of cannabis use in university students. The authors concluded that EI was a protective factor against cannabis consumption, which also reduced the influence to use exerted by the peer group.

This research aims to analyse the involvement of EI with respect to cannabis use in adolescence, and combines the assessment of trait and ability EI factors in order to provide a complete understanding of their role in the use of this substance. In addition, this study is one of the first to analyze the incremental and predictive validity of trait and ability EI with respect to other classical variables of protection, such as coping styles and assertiveness in relation to the cannabis use habit in adolescents (“consumption of cannabis ever in life”, “frequency of cannabis use in the last 12 months”, “number of joints per week during the last month”, “cannabis use when offered by friends”). Finally, this research adds relevant information on the youngest consumers’ habits (12–13 years) in order to guide future preventive interventions. The study hypotheses based on the previous literature are detailed below:

**Hypothesis** **1** **(H1).** *The factors of trait EI (emotional repair and emotional clarity) and ability EI (emotional perception, emotional facilitation, emotional understanding, and emotional regulation), styles of active and social coping, as well as assertive behavior will correlate negatively and significantly with the variables of cannabis use. However, avoidant coping style and emotional attention factors will correlate positively and significantly with them.*

**Hypothesis** **2** **(H2).** *Trait and ability EI factors will be associated with the variables of cannabis use by taking into account other classical variables of protection such as coping styles and assertiveness, which will be maintained once age and gender are controlled for.*

## 2. Materials and Methods

### 2.1. Study Sample

Ten Spanish schools in the region of Cantabria participated in this research. Following the proportion present in the reference population, stratified random sampling was carried out according to the type of schools (public or private) and its location in a rural or urban environment, assuring a representation of diverse socio-economic contexts. The participation rate of the initially selected schools was 66.6% due to the heavy workload of some of them. Initially, a total sample of 844 Spanish students was obtained. Questionnaires of adolescents older than 16 years (N = 21) as well as the incomplete ones (N = 24) were excluded. The final sample consisted of 799 participants (51.8% men and 48.2% women) with ages between 12 and 16 years (12–13 years, 38.2%; 14–16 years, 61.8%; M = 14.49; SD = 1.17). The adolescents belonged to private (51.4%) and public schools (48.6%) located in urban (64%) and rural (36%) contexts.

### 2.2. Measures

Dependent variables: Cannabis use questionnaire. Four items from the Spanish Survey on Drug use in Secondary School Students (ESTUDES) [3], which is a biannual survey that collects the drug habit in adolescents, were adapted: (1) Have you ever used cannabis? (Yes, I have used it/No, I have not used it); (2) On how many days have you used cannabis in the last 12 months? (1–7, 8–14, 15–21, 22–28, 29–35, 36–42, 43–49, 50 or more); (3) How many joints did you smoke per week in the last month? (1–2, 3–4, 5–6, 7–8, 9–10, 11–12, 13–14, 15–16, 17–18, 19–20, 21 or more); (4) If any of your friends offered you cannabis, would you consume it? (Yes, I would consume it/No, I would not consume it).

Control variables: Sociodemographic data questionnaire. The gender (female/male) and age of the schoolchildren (12–13/14–16 years) were asked.

Independent variables:

Brief COPE ([43]; validated in the Spanish population by Crespo and Cruzado, [44]). This self-report measures the different forms of stress response. It consists of 28 items grouped into 14 subscales evaluated on a four-point Likert scale (1 = Strongly disagree; 4 = Strongly agree). For this study, a factor analysis was carried out whose theoretical solution was adjusted to the model proposed by Morán, Landero, and González [45]. The spiritual coping dimension was excluded since it did not obtain a significant factor load. The dimensions used in this study were: (a) Active coping composed by the subscales of planning, humor, acceptance, cognitive reappraisal, self-distraction, and active coping attitude; (b) social coping, with the subscales of emotional support and instrumental support to ask for advice or help; (c) unproductive or avoidance coping with the subscales of behavioral disconnection, denial, substance use and self-blame. Cronbach’s alpha coefficient for this study was 0.72, 0.67, and 0.70, respectively.

Assertive Behavior self-report (ADCAS; [18]). This instrument measures aspects related to the expression of feelings, respect for values, and desires or preferences of oneself and of others. In this study, only the self-assertiveness subscale was used, which is made up of 20 items (e.g., “I find hard to say no when they ask me to do something I don’t want to do”), evaluated using a 4-point Likert scale (1 = Strongly disagree; 4 = Strongly agree). Cronbach’s alpha coefficient for this study was 0.84.

Trait Meta-Mood Scale (TMMS; [46]; validated by Salguero et al. [47]). This self-report measures trait EI. It is made up of 24 items that are subdivided into three subscales: (a) Emotional attention (e.g., “I pay a lot of attention to how I feel”); (b) emotional clarity (e.g., “I can’t make sense out of my feelings”); (c) emotional repair (e.g., “when I become upset, I remind myself of all the pleasures in life”). It has a 5-point Likert-type scale (1 = Disagree at all; 5 = Totally agree). For this study, Cronbach’s alpha was 0.87, 0.85, and 0.82 for emotional attention, emotional clarity, and emotional repair, respectively.

Test of Emotional Intelligence by the Fundación Botín for Adolescents (TIEFBA; original Spanish validation by Fernández-Berrocal et al. [48]). This maximum performance test evaluates the level of skill in the four emotional abilities of the theoretical model of Mayer and Salovey [34]. It is made up of 143 items that pose emotional situations by means of eight short stories involving adolescent characters and reporting four scores: (a) Emotional perception (e.g., “To what extent do you think Rubén feels these emotions?”); (b) emotional facilitation (e.g., “To what extent feeling like this will help Rubén to prepare a complicated kitchen recipe with his mother?”); (c) emotional understanding (e.g., “What Rubén can be thinking to feel like this?”); (d) emotional regulation (e.g., “What can Rubén do to feel better during the class?”). It has a 5-point Likert-type scale (1 = Disagree at all; 5 = Totally agree). For this study, Cronbach’s alpha was 0.86, 0.78, 0.80, and 0.76 for emotional perception, emotional facilitation, emotional understanding, and emotional management, respectively.

### 2.3. Procedure

The development of the research plan for the present study was approved by the Academic Commission for Doctoral Studies of the University of Cantabria. This research is governed by the principles set out in the Declaration of Helsinki [49] and has the written informed consent of all participants. First, an informative document was issued to the schools, families, and students to obtain their signed authorization prior to participating in the study. The researchers were together with the students and their teachers, while the instruments were administered in their own classroom at the beginning of the school year. Anonymity was ensured using numerical codes. At the end, researchers kept their answers in a sealed envelope. Students were asked about habits related to cannabis use, trait EI, coping strategies, and assertiveness through a questionnaire and they also filled an EI maximum performance test. The time allowed to complete the questionnaire was two sessions of 50-min on alternative days.

### 2.4. Statistical Analyses

Data were analyzed using SPSS version 24.0. Firstly, a descriptive analysis showed the prevalence of cannabis consumption (Table 1). Secondly (Hypothesis 1), a point bias correlation analysis was carried out with the study variables (Table 2). Thirdly, (Hypothesis 2), binomial regression models for the calculation of the prevalence ratios of cannabis were performed [50], based on the independent variables used (emotional attention, emotional clarity, emotional repair, emotional perception, emotional facilitation, emotional understanding and emotional regulation, active coping, social coping, avoidance coping, and assertiveness) to observe their association with the dependent variables once age and gender were controlled for (1. Have you ever used cannabis?; 2. On how many days have you used cannabis in the last 12 months?; 3. How many joints did you smoke per week in the last month?; 4. If any of your friends offered you cannabis, would you consume it?). Dependent variables 1 and 4 were dichotomous, and variables 2 and 3 were dichotomized by the median before being entered into the regression analyses. The factors that significantly correlated were entered into the regression models. In addition, the backward steps method was used to extract the independent variables one by one until the factors were significant to form each regression model (at least for *p* < 0.05). In this study, the final regression models are presented to synthesize the amount of information.

## 3. Results

Table 1 shows the prevalence of cannabis use. First of all, it should be noted that from the total sample (N = 799), approximately a fifth claimed to have tried cannabis at some point (22.3%). Among the users (N = 179), about three-quarters (N = 74.9%) had smoked for 40 days or more in the past year. Likewise, approximately half of the consumers (51.4%) had smoked 10 or more joints per week during the last month. Regarding the total sample (N = 799), a fifth (20.5%) claimed to have used cannabis when offered by friends. Regarding the sociodemographic variables in the total sample, 18.3% of the women stated that they had ever used cannabis, while the prevalence in men was somewhat higher (26.4%). Regarding the age groups, approximately 10% of adolescents aged 12–13 years stated that they had smoked at least once (13.9%); this percentage was higher in the older group (27.3%).

The results are detailed below according to the study hypotheses.

In relation to Hypothesis 1, significant negative correlations were observed between the predictor variables and the cannabis use variables, with the exception of the emotional attention and avoidant coping factors, which were positively correlated with some of these variables. The correlations showed a low effect size [51]. The descriptive results of the predictor variables as well as the correlation with the consumption-dependent variables are presented in Table 2.

Taking into account Hypothesis 2, to study the effect of independent variables regarding cannabis use, binomial regression analyses were carried out controlling for gender and age (Table 3). Firstly, when we took “cannabis use at some time” as a dependent variable, the age and independent variables of emotional repair, emotional perception, assertiveness, and avoidance coping formed the model. Among these, the highest PR were found for the avoidant coping factor (PR = 1.03; 95% CI (1.01–1.05)), this being similar to those of emotional repair (PR = 0.96; 95% CI (0.94–0.97)), emotional perception (PR = 0.97; 95% CI (0.96–0.98)), and assertiveness (PR = 0.97; 95% CI (0.95–0.98)). Additionally, adolescents in the 14–16 group had a prevalence of use 1.66 times higher than that in the 12–13 group. Secondly, when we took “frequency of cannabis use per year” as a dependent variable, the independent variables of emotional repair (PR = 0.96; 95% CI (0.93–0.98)) and active coping formed the model (PR = 0.96; 95% CI (0.94–0.99)). Thirdly, when we took the “number of weekly joints during the last month” as a dependent variable, the independent variable of emotional clarity (PR = 1.01; 95% CI (1.01–1.02)) formed the model. Finally, when we took “cannabis use when offered by friends” as a dependent variable, the age and independent variables of emotional attention, emotional perception, emotional facilitation, and assertiveness became part of the model. Among these, the highest PR were found for the emotional attention factor (PR = 1.03; 95% CI (1.01–1.06)), this being similar to those of emotional perception (PR = 0.98; 95% CI (0.96–0.99)), emotional facilitation (PR = 0.97; 95% CI (0.95–0.98)), and assertiveness (PR = 0.97; 95% CI (0.95–0.98)). Additionally, adolescents in the 14–16 group had a prevalence of use 2.12 times higher than that in the 12–13 group.

As can be seen, among the sociodemographic variables of gender and age, the gender variable was not significant and only the age was significant for “cannabis use at some time” and “cannabis use when offered by friends” dependent variables (Table 3). Therefore, results were then stratified according to age (12–13 and 14–16 years age group) to observe how personal protective factors influence both groups. First, the model created for the “cannabis use at some time” dependent variable in the 12–13 years age group (Table 4) was formed by the independent variables of emotional repair (PR = 0.94; 95% CI (0.90–0.98)) and emotional perception (PR = 0.95; 95% CI (0.93–0.97)). Likewise, the model created for this variable in the 14–16 years age group (Table 5) was formed by emotional repair, emotional perception, assertiveness, and avoidant coping independent variables. The highest PR were found for avoidant coping (PR = 1.04; 95% CI (1.02–1.06)), followed by emotional perception (PR = 0.97; 95% CI (0.96–0.98)), assertiveness (PR = 0.97; 95% CI (0.95–0.98)), and emotional repair (PR = 0.96; 95% CI (0.94–0.98)). Secondly, the model created for the dependent variable “cannabis use when offered by friends” in the group 12–13 years (Table 6) was formed by the independent variables of emotional perception (PR = 0.95; 95% CI (0.93–0.98)), emotional facilitation (PR = 0.96; 95% CI (0.94–0.99)), and assertiveness (PR = 0.95; 95% CI (0.90–0.99)). In this line, the model created for this variable in the 14–16 years age group (Table 7) was formed by emotional attention, emotional perception, emotional facilitation, and assertiveness independent variables. The highest PR were found for emotional attention (PR = 1.03; 95% CI (1.01–1.06)), followed by emotional perception (PR = 0.97; 95% CI (0.97–1.00)), emotional facilitation (PR = 0.97; 95% CI (0.95–0.98)), and assertiveness (PR = 0.97; 95% CI (0.95–0.99)).

## 4. Discussion

The results of this study show that the prevalences of cannabis use in adolescents broadly coincide with the data reported in the last Spanish Survey on Drug Use in Secondary School Students [3]. This fact has important implications at the prevention level and exalts the need for intensive work at the beginning of Compulsory Secondary Education and even in the the last years of Primary Education.

The present research analysed the influence of coping styles and assertiveness together with the effects of trait EI and ability EI on cannabis consumption habits in adolescence. First, it should be noted that the first working hypothesis was completely fulfilled. Our results provide new information on the role of these factors in the school population. Based on the data obtained, it is observed that the style of active and social coping, as well as assertiveness and the factors of trait EI (emotional clarity and emotional repair) and ability EI (emotional perception, emotional facilitation, emotional understanding, and emotional regulation) appear to protect against some cannabis consumption behaviors; on the other hand, avoidant coping style and high emotional attention are shown as risk factors. Considering the size of the effect of the correlations between the predictor and the dependent variables, it should be noted that these correlations were weak [51], as has been observed in previous studies on drug use in adolescents [52,53]. Secondly, the second hypothesis of the study was partially fulfilled. Once the age and gender of the participants were taken into consideration, all the factors of trait EI (emotional attention, emotional clarity, and emotional repair) and only the ability EI factors of emotional perception and emotional facilitation helped explain the consumption habits alongside coping styles and assertiveness. Taking into account the age groups (12–13/14–16 years), the results showed that the perceived ability to repair negative emotional states and the ability to perceive one’s own and other’s emotions adequately explained cannabis use at some point in life in both age groups. On the other hand, assertiveness and avoidant coping styles predicted higher probability of consumption in the older group. In the same way, assertiveness and the abilities to appropriately perceive and understand emotions were explanatory of cannabis use when offered by friends in both adolescent age groups. Likewise, attentiveness to one’s own emotions acted as a risk factor only in the oldest age group. This pattern of results suggests that emotional components have value when it comes to improving thought patterns [54]. Moreover, they are an important preventive factor for risk behaviors such as cannabis use in the adolescent stage.

In this study, active coping with strategies such as planning, humour, acceptance, cognitive reappraisal, etc., was related to lower frequency of cannabis use in the last year. This result is aligned with other investigations in which a negative association was found between active coping and behaviors related to the use of addictive substances in the adolescent population [17,24,25]. A recent longitudinal study has revealed that greater active coping in middle adolescence prospectively predicts lower cannabis use in late adolescence [55]. These data suggest that active coping makes it possible to control stressors through a broader repertoire of responses [23]. This aspect could explain responsible decision-making by helping to prevent the use of cannabis at an early age. On the other hand, the avoidant coping style (behavioral disconnection, denial, self-blame, etc.) predicted a greater probability of ever having tried cannabis. This result is in accordance with recent research [25,26,27], which found a positive association between this coping style and a greater probability of alcohol and cannabis consumption, as well as a greater quantity and frequency of use of these substances in adolescents. In this study, avoidance coping style was associated with cannabis use at some point in life for the oldest adolescent age group (14–16 years). The literature has highlighted that the use of cannabis begins at around the age of 14 [10] and may be a way of regulating unpleasant emotional states due to a lack of personal resources [40,56], which could facilitate maladjustment and avoidance behaviors in the face of problems in the older group [57]. It should be mentioned that, despite the fact that social coping correlated negatively and significantly with some variables of cannabis use, it did not have high explanatory power when considering the other personal factors. Despite this, the obtained results suggest that clinical and educational interventions could be beneficial in the development of positive coping strategies and also contribute to the reduced use of harmful substances such as cannabis at these ages.

Regarding the role of assertiveness in this study, it should be noted that it was associated with cannabis use at some point in life. This result was the same for the older group (14–16 years) and for the use of cannabis when offered by friends. In agreement with the previous research [10,58], our results contribute to the proposition that assertive responses allow the effective management of situations related to the use of drugs, thus reinforcing an adolescent’s ability to withstand peer pressure. The influence of the peer group during this period has frequently been suggested as one of the factors most associated with cannabis use [59]. Based on the previous research [60], non-normative peers are likely to promote adolescent cannabis use, which in turn can be seen as a maladaptive means of constructing and establishing personal and social identity, especially in the older group (14–16 years). For this reason, it could be said that having assertiveness skills has a favourable influence when strong resistance to peer pressure is required, as this is something that reduces young people’s vulnerability to the risk of consumption at these ages.

In relation to trait EI, a greater perceived ability to repair negative emotional states by prolonging positive ones predicted a lower probability of ever having used cannabis in both age groups (12–13/14–16 years). Likewise, a greater perceived ability to understand and repair one’s own negative emotional states was associated with a lower quantity and frequency of cannabis use in adolescents. The findings of this study are aligned with others in which a negative relationship was found between scores in emotional repair and clarity and the use of addictive substances in adolescents and university students [38,40,56,61]. Our results support the idea that adolescents who better understand their emotions and have a better implementation of strategies for regulating negative emotional states are less likely to use cannabis as a way to mitigate aversive emotional states [40,56]. In this study, on the other hand, a high level of emotional attention predicted a greater probability of consumption due to peer pressure, a result that was the same for the older group (14–16 years). These data are in line with what was found in previous literature: Higher levels of emotional attention were associated with alcohol, tobacco, and cannabis abuse in adolescents and university students [40,56,62]. In this sense, emotional attention has shown positive associations with anxiety, depression, and misadjusted coping strategies such as ruminative thinking in young people [63,64,65]. Given that the onset of cannabis use occurs at around the age of 14 [3], it could make sense that emotional attention acts as a facilitator of consumption in middle adolescence, a period in which the search for personal identity, distancing from family values, and the need for peer acceptance are also accentuated [66].

Lastly, it is worth highlighting the role of ability EI. In this study, a greater ability to adequately perceive emotions predicted a lower probability of ever having used cannabis in both age groups (12–13/14–16 years). Likewise, greater perception and emotional facilitation were associated with a lower probability of cannabis use due to peer pressure, a result that was maintained for both age groups. These data coincide with a study by Brackett et al. [42], which observed negative associations between emotional abilities and the consumption of alcohol and cannabis among a peer group. As in previous research [67], our results support the idea that a greater difficulty in perceiving and using emotions hinders decision-making and cognitive performance, which could pose a problem for adolescents when identifying peer pressure and managing discrepancies between their motivations and those of others. On the other hand, one reason that could explain the greater use of these two skills (emotional perception and emotional facilitation) with respect to understanding and regulating emotions could be the hierarchical arrangement of the four-branch model of Salovey and Mayer [34], emotional perception and emotional facilitation abilities are preceded by understanding emotional processes and awareness of their variations so as to regulate emotional information in order to adapt appropriately to different contexts [68]. Based on advances in neuroscience, the frontal lobes and other areas of the brain are known to mature at around the age of 18–20 [69,70]. In this way, the maturation of the brain under normal conditions implies the process of myelination, which favours interconnectivity and increases the capacity to understand and consciously regulate emotions and behavior. Therefore, it is possible that the young age of the adolescents who participated in this research (12–16 years) justifies a greater use of emotional perception and emotional facilitation skills compared to the other types of EI, which mature later.

## 5. Limitations of the Study

It should be noted that this study has certain methodological limitations. Firstly, the reliability obtained for social coping dimension of the Brief Cope scale was 0.67. However, we would like to clarify that the specialized literature accepts 0.5 or higher as acceptable on short scales [71]. Likewise, we would like to appoint that in this study we obtained higher alpha indices than in the original validation of the instrument [43]. Secondly, it is important to highlight the cross-sectional nature of this research. Therefore, future works should continue to corroborate results found by means of prospective designs that allow causal relationships between the studied variables to be inferred. Despite this, the present study facilitates empirical data regarding the effect of personal variables of protection related to cannabis use in adolescence and highlights the necessary inclusion of EI within them. However, future research could control ethnicity/race or family consumption variables as covariates.

## 6. Conclusions

This study expands on previous evidence regarding the protective value that is exerted by EI, active coping styles, and assertiveness regarding cannabis use in adolescence. Our results are aligned with the contributions of various authors who propose the promotion of resources and personal competencies in order to facilitate well-being and resistance to risk situations such as drug use [72]. The data obtained in this study are in line with scientific advances that highlight the training of social and emotional skills from different preventive contexts such as educational, family or community [73]. Thus, despite the fact that the risk variables related to cannabis use are numerous and difficult to modify, the skills included in this study can be improved and learned by acting as protective factors for cannabis use at an early age. In this sense, it can be said that adolescents who are more capable of perceiving and using emotions, attending to them in a moderate manner, and understanding and repairing their emotions adequately when they are negative, are less likely to use cannabis. Likewise, it is worth highlighting the protective value of active coping and assertiveness when it comes to avoiding risks and dealing with group pressure to consume this substance. Therefore, we consider that this research offers relevant information that could guide the design of clinical and educational interventions focused on preventing harmful habits at an early age.

## Figures and Tables

**Table 1:** Descriptive analysis of cannabis use.

Cannabis Consumption Variables	Response Categories	Percentage	N
Have tried cannabis before	Yes	22.3	179
	No	77.7	620
Frequency of consumption per year	≥40 days	74.9	134
	<40 days	25.1	45
Number of weekly units	≥10	51.4	92
	<10	48.6	87
Used when offered by friends	Yes	20.5	161
	No	79.5	638
Use by gender	female yes	18.3	73
	female no	81.8	327
	male yes	26.4	106
	male no	73.6	293
Use by age	12–13 years old yes	13.9	41
	12–13 years old no	73.7	255
	14–16 years old yes	27.3	137
	14–16 years old no	58.9	366

**Table 2:** Descriptive and biserial point correlation analysis of personal factors with respect to cannabis use variables.

Personal Factors and Cannabis Variables	M	SD	Have Tried Cannabis Before	Frequency of Consumption per Year	Number of Weekly Units	Used When Offered by Friends
1. Emotional Attention (TMMS)	24.3	7.4	0.25 **	0.23 **	0.08	0.29 **
2. Emotional Clarity (TMMS)	22.3	7.2	−0.28 **	−0.23 **	−0.21 **	−0.30 **
3. Emotional Repair (TMMS)	24.8	7.4	−0.32 **	−0.25 **	−0.10	−0.26 **
4. Emocional Perception (TIEFBA)	100.6	13.1	−0.36 **	−0.17 *	−0.25 **	−0.35 **
5. Emotional Facilitation (TIEFBA)	104.4	14.8	−0.34 **	−0.22 **	−0.21 **	−0.34 **
6. Emotional Understanding (TIEFBA)	104.2	14.4	−0.29 **	−0.08	−0.20 **	−0.24 **
7. Emotional Regulation (TIEFBA)	98.5	8.9	−0.28 **	−0.08	−0.12	−0.17 *
8. Assertiveness	41.2	10.2	−0.25 **	−0.11	−0.13	−0.32 **
9. Avoidant coping	16.3	5.5	0.26 **	0.18 *	25 **	0.14
10. Active coping	32.6	6.2	−0.17 *	−0.26 **	−17 *	−0.13
11. Social coping	29.8	5.8	−0.16 *	−0.17 *	−0.11	−0.22 **

Notes: * = *p* < 0.05; ** = *p* < 0.01; M = mean; SD = standard deviation; (TMMS) = Trait Meta-Mood Scale; (TIEFBA) = Test of emotional intelligence by Fundación Botín for adolescents.

**Table 3:** Binomial regression analysis of personal protective factors controlled by age and gender regarding cannabis use.

Cannabis Variables and Personal Factors	B	SE	Wald	PR	CI 95% of the PR
**Have tried cannabis**					
Age	0.51	0.17	8.906 **	1.66	1.19/2.31
Emotional Repair (TMMS)	−0.04	0.01	16.41 **	0.96	0.94/0.97
Emotional Perception (TIEFBA)	−0.03	0.00	32.91 **	0.97	0.96/0.98
Assertiveness	−0.03	0.01	17.59 **	0.97	0.95/0.98
Avoidant coping	0.03	0.01	9.57 **	1.03	1.01/1.05
**Frequency of consumption per year**					
Emotional Repair (TMMS)	−0.04	0.01	11.86 **	0.96	0.93/0.98
Active coping	−0.03	0.01	9.75 **	0.96	0.94/0.99
**Number of weekly units**					
Emotional Clarity (TMMS)	−0.13	0.00	13.80 **	1.01	01.01/1.02
**Usage when offered by friends**					
Age	0.75	0.22	11.22 **	2.12	1.37/3.29
Emotional Attention (TMMS)	0.03	0.01	6.81 **	1.03	1.01/1.06
Emotional Perception (TIEFBA)	−0.02	0.00	10.16 **	0.98	0.96/0.99
Emotional Facilitation (TIEFBA)	−0.03	0.00	24.32 **	0.97	0.95/0.98
Assertiveness	−0.03	0.01	10.38 **	0.97	0.95/0.98

Notes: ** = *p* < 0.01; B = coefficient; SE = standard error; PR = prevalence ratios; CI = confidence interval; (TMMS) = Trait Meta-Mood Scale; (TIEFBA) = Test of emotional intelligence by the Fundación Botín for adolescents.

**Table 4:** Binomial regression analysis based on the 12–13 years age group.

Cannabis Variables and Personal Factors	B	SE	Wald	PR	CI 95% of the PR
Have tried cannabis					
Emotional Repair (TMMS)	−0.06	0.02	7.45 **	0.94	0.90/0.98
Emotional Perception (TIEFBA)	−0.50	0.01	16.60 **	0.95	0.93/0.97

Notes: ** = *p* < 0.01; B = coefficient; SE = standard error; PR = prevalence ratios; CI = confidence interval; (TMMS) = Trait Meta-Mood Scale; (TIEFBA) = Test of emotional intelligence by the Fundación Botín for adolescents.

**Table 5:** Binomial regression analysis based on the 14–16 years age group.

Cannabis Variables and Personal Factors	B	SE	Wald	PR	CI 95% of the PR
Have tried cannabis					
Emotional Repair (TMMS)	−0.04	0.01	11.48 **	0.96	0.94/0.98
Emotional Perception (TIEFBA)	−0.02	0.00	22.34 **	0.97	0.96/0.98
Assertiveness	−0.03	0.01	15.59 **	0.97	0.95/0.98
Avoidant coping	0.04	0.01	12.53 **	1.04	1.02/1.06

Notes: ** = *p* < 0.01; *B* = coefficient; SE = standard error; PR = prevalence ratios; CI = confidence interval; (TMMS) = Trait Meta-Mood Scale; (TIEFBA) = Test of emotional intelligence by the Fundación Botín for adolescents.

**Table 6:** Binomial regression analysis based on the 12–13 years age group.

Cannabis Variables and Personal Factors	B	SE	Wald	PR	CI 95% of the PR
Usage when offered by friends					
Emotional Perception (TIEFBA)	−0.05	0.01	9.63 **	0.95	0.93/0.98
Emotional Facilitation (TIEFBA)	−0.04	0.01	6.50 *	0.96	0.94/0.99
Assertiveness	−0.05	0.02	5.87 *	0.95	0.90/0.99

Notes: * = *p* < 0.05; ** = *p* < 0.01; *B* = coefficient; SE = standard error; PR = prevalence ratios; CI = confidence interval; (TMMS) = Trait Meta-Mood Scale; (TIEFBA) = Test of emotional intelligence by the Fundación Botín for adolescents.

**Table 7:** Binomial regression analysis based on the 14–16 years age group.

Cannabis Variables and Personal Factors	B	SE	Wald	PR	CI 95% of the PR
Usage when offered by friends					
Emotional Attention (TMMS)	0.03	0.01	6.49 **	1.03	1.01/1.06
Emotional Perception (TIEFBA)	−0.02	0.00	4.91 *	0.98	0.97/1.00
Emotional Facilitation (TIEFBA)	−0.03	0.00	20.27 **	0.97	0.95/0.98
Assertiveness	−0.03	0.01	6.45 **	0.97	0.95/0.99

Notes: * = *p* < 0.05; ** = *p* < 0.01; *B* = coefficient; SE = standard error; PR = prevalence ratios; CI = confidence interval; (TMMS) = Trait Meta-Mood Scale; (TIEFBA) = Test of emotional intelligence by the Fundación Botín for adolescents.

## Data Availability

The data from this study are not published due to the fact that the research group continues to work with them. However, they can be requested through the authors.
